# Phylogenetic placement and microthrix pattern of *Paranybelinia otobothrioides* Dollfus, 1966 (Trypanorhyncha) from krill *Nyctiphanes simplex* Hansen, 1911

**DOI:** 10.1016/j.ijppaw.2019.08.006

**Published:** 2019-08-27

**Authors:** José Raúl Morales-Ávila, Jaime Gómez-Gutiérrez, Norma Y. Hernandez-Saavedra, Carlos J. Robinson, Harry W. Palm

**Affiliations:** a Ecología y Conservación de La Vida Silvestre A.C. (ECOVIS), Marcelo Rubio, entre Oaxaca y Jalisco 3530, La Paz, Baja California Sur, 23060, Mexico; b Instituto Politécnico Nacional, Centro Interdisciplinario de Ciencias Marinas (CICIMAR), Departamento de Plancton y Ecología Marina, Avenida IPN s/n, La Paz, Baja California Sur, 23096, Mexico; c Laboratorio de Genética Molecular, Centro de Investigaciones Biológicas del Noroeste S.C. (CIBNOR), Mar Bermejo 195, La Paz, Baja California Sur, Mexico; d Universidad Nacional Autónoma de México, Instituto de Ciencias del Mar y Limnología (ICMyL), Ciudad Universitaria, Mexico City, DF, 04500, Mexico; e University of Rostock, Faculty of Agricultural and Environmental Sciences, Aquaculture and Sea-Ranching, Justus-von-Liebig-Weg 6, 18059, Rostock, Germany

**Keywords:** Cestoda, Paranybeliniidae, Blastocyst, Surface ultrastructure, Microtriches, ssrDNA

## Abstract

Plerocerci of the monotypic *Paranybelinia otobothrioides* were found parasitizing the subtropical neritic krill *Nyctiphanes simplex* in the Gulf of California, Mexico. The plerocerci were recovered from two microhabitats of the intermediate host, typically embedded inside the digestive gland (hepatopancreas) or rarely in the hemocoel. The morphology of the simple, single-layered blastocyst surrounding the entire scolex is unique within the Trypanorhyncha by having four large funnel-like pori or openings possibly with feeding and/or excretory function. One of the openings is located anteriorly and three at the posterior end. Scolex surface ultrastructure shows hamulate and lineate spinitriches covering the bothrial surface, capilliform filitriches at the anterior scolex end and on the scolex peduncle, and short papilliform filitriches on the long appendix. This pattern resembles that of species of the Tentaculariidae; but differs in that the hamulate spinitriches, which appear lineate at the bothrial margins, densely cover the entire distal bothrial surface. Tegumental grooves are present on the posterior bothrial margin, lacking spinitriches. *Paranybelinia otobothrioides* and *Pseudonybelinia odontacantha* share the following unique combination of characters: two bothria with free lateral and posterior bothrial margins, homeoacanthous homeomorphous armature, tegumental grooves, the distribution of the hamulate spinitriches, and the absence of prebulbar organs. Both genera infect euphausiids as intermediate hosts. Sequence data of the partial ssrDNA gene place *Pa. otobothrioides* sister to the family Tentaculariidae, and the Kimura two-parameters (K2P) distance between *Pa. otobothrioides* and species of the family Tentaculariidae ranged from 0.027 to 0.039 (44-62 nucleotide differences). These data suggest both species be recognized in a family, the Paranybeliniidae, distinct from, albeit as sister taxon to, the Tentaculariidae. High prevalence of infection (<14%) and ontogenetic changes of *Pa. otobothrioides* support *N. simplex* as a required intermediate host and suggest a zooplanktophagous elasmobranch as final host in the Gulf of California.

## Highlights

• First description of the microthrix pattern of *Paranybelinia otobothrioides* Dollfus (1966) larva.
• The krill *Nyctiphanes simplex* is the first known host of larvae of *P. otobothrioides*.
• Blastocyst morphology surrounding the scolex is unique within the Trypanorhyncha.
• SsrDNA sequence places *P. otobothrioides* as sister taxon to the Tentaculariidae.
• We emmend the superfamily diagnosis in the most recent classification.

## Graphical abstract

**Figure:**
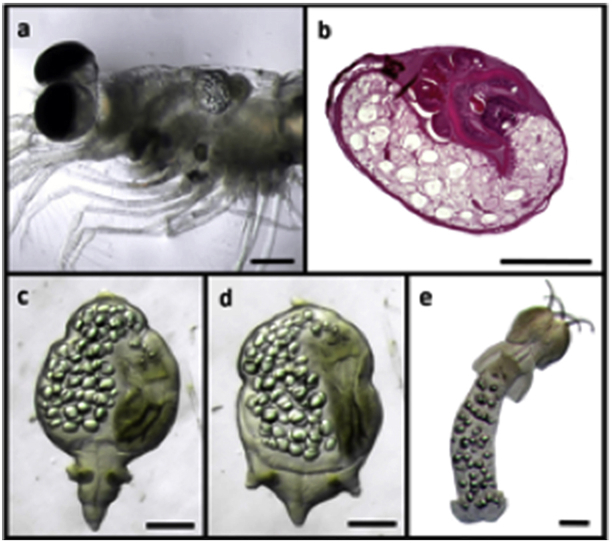

## 1 Introduction

Cestodes of the order Trypanorhyncha are distributed worldwide. Over 300 species infect the stomach and intestine of their elasmobranch final hosts and their metacestodes infect marine invertebrates (mostly zooplanktonic) and teleost fish (Campbell and Beveridge, 1994; Palm, 1999, 2004; 2008; Caira and Jensen, 2017). However, while most trypanorhynchs have been reported from teleost and elasmobranch fish (Palm, 2004), there are several reports of metacestodes infecting marine zooplankton (Shipley and Hornell, 1906; Anantamaran, 1963; Dollfus, 1966, 1967; Grabda, 1968; Slankis and Shevchenko, 1974; Shimazu, 1975, 1982, 1999; 2006; Reimer, 1977; Mauchline, 1980; Mooney and Shirley, 2000; Gómez-Gutiérrez et al., 2010, 2017; González-Solís et al., 2013). Consequently, the morphology of trypanorhynch metacestodes, zoogeographical distribution and their transmission dynamics during the early life cycle stages is scarcely studied.

Dollfus (1966) described the trypanorhynchs *Pseudonybelinia odontacantha* Dollfus, 1966 and *Paranybelinia otobothrioides* Dollfus, 1966 recovered off the Cape Verde Islands, Africa. Both species showed unique scolex features with presence of the so-called bothrial pits and a homeoacanth armature, leading to the erection of an own family Paranybeliniidae. Because both species were originally recovered as metacestodes free from marine plankton in the presence of unidentified euphausiids, their zooplankton paratenic/intermediate hosts and life cycles remained completely unknown. After Dollfus's finding, *Ps. odontacantha* was reported infecting the euphausiid *Euphausia recurva* Hansen, 1905 from the East China Sea (Shimazu, 1982, 2006), whereas, *Pa. otobothrioides* has not been recorded since then (Dollfus, 1966; Palm, 2004, 2008). Remarkably, the *Pa. otobothrioides* type material of the latter kept in the collection of the Museum National d'Histoire Naturelle, Paris, does not allow further taxonomic revision or re-description (Campbell and Beveridge, 1994; Palm, 2008). Consequently, the validity of the family Paranybeliniidae as well as its relationship with other species within the superfamily Tentacularioidea is still under debate (Palm, 2008). In this regard, the scolex morphology, especially the number of bothria, presence or absence of a blastocyst in metacestodes, prebulbar organs, bothrial pits as well as the arrangement of the hooks around the tentacles are characters typically used to distinguish the species in the order Trypanorhyncha (Palm, 2004).

The analysis of the surface ultrastructure, especially the characterization and distribution pattern of microtriches of the bothria and scolex peduncle, observed with scanning electron microscopy (SEM) also provide relevant taxonomic information within the trypanorhynchs (Faliex et al., 2000; Chervy, 2009), though most of these taxonomic characters are unknown in *Pa. otobothrioides* (Palm, 1995, 2004, 2008). The systematic position of *Pa. otobothrioides* has been shifting between the families Tentaculariidae and Otobothriidae due to the incomplete morphological information, which lacks description of the surface ultrastructure, strobilar characters and reproductive system of the adults (Schmidt, 1986; Campbell and Beveridge, 1994; Palm, 1995, 1997; 2004; Beveridge et al., 1999). In this context, *Pa. otobothrioides* was allocated close to the Tentaculariidae on the bases of the scolex morphology resembling the genus *Nybelinia*, the homeoacanthous armature as well as on the assumption that its plerocercoid lacks a blastocyst (Schmidt, 1986; Campbell and Beveridge, 1994; Beveridge et al., 1999). In contrast, this species was moved to the Otobothrioidea due to the possession of two bothria with bothrial pits (Palm, 1995, 1997, 2004). More recently, Palm (2008) confirmed that the species *Ps. odontacantha* possesses tegumental grooves on the posterior bothrial margin and that its microthrix pattern with hamulate spinitriches resembles that observed in the Tentaculariidae, moving the Paranybeliniidae into the superfamily Tentacularioidea. Detailed description of new material of *Pa. otobothrioides* is required to investigate some morphological characters used for the diagnosis of the family Paranybeliniidae and to evaluate its systematic position.

We describe the internal morphology of the plerocerci of *Pa. otobothriodes* recovered from the subtropical neritic krill *Nyctiphanes simplex* Hansen, 1911 in the Gulf of California, Mexico. Additionally, we describe the external ultrastructure of the blastocyst, the microthrix pattern of the scolex, anatomy and its phylogenetic placement within the order Trypanorhyncha based on a nearly complete ssrDNA fragment. Implications for the life cycle, biology and current trypanorhynch cestode taxonomical classification, including the family Paranybeliniidae, are discussed.

## 2 Materials and methods

### 2.1 Sampling and light microscopy

Zooplankton samples were collected during four oceanographic cruises (January and July 2007, August 2012 and June 2013) on board the R/V El Puma (UNAM) at a total of 90 sampling stations in the Gulf of California (24–32° N, 109–115° W). Zooplankton was quantitatively sampled with oblique tows from the surface to 280 m water depth with standard Bongo nets, one with 333 μm and another with 500 μm mesh size. Nets were equipped with a calibrated digital flowmeter (General Oceanics, Miami, FL) to estimate volume of filtered seawater using standard methods (Smith and Richardson, 1977). Only zooplankton samples collected with the 500 μm net were preserved with 96% non-denatured ethanol on board analyses. All krill were sorted and analyzed for parasite infection. Euphausiids were additionally collected during August 2012 and June 2013 cruises with a 1-m opening diameter net (500 μm) towed at night (<40 m depth) to observe and measure them alive with a stereoscope (SV11, Carl Zeiss equipped with a micrometer). Species of krill were identified using standard taxonomic keys (Baker et al., 1990; Brinton et al., 2000). Live krill specimens infected with endoparasites were photographed with a digital Cannon G11 camera (3.3 megapixels resolution). Each krill specimen was dissected with entomological needles to record the presence/absence of helminths in their cephalothorax, focusing on the gonad, the hepatopancreas (digestive gland) and the trunk of juvenile and adult krill.

Plerocerci of *Pa. otobothrioides* (Tentacularioidea, Paranybeliniidae) were found parasitizing the subtropical krill *N. simplex*. The blastocysts containing the scolices were removed from the hepatopancreas of live parasitized *N. simplex* specimens observed on board, washed in saline solution, and fixed in 10% formalin or 70% ethanol for morphological analyses. Additionally, plerocerci from 96% ethanol-fixed hosts were used for subsequent molecular analyses. Specimens prepared as whole mounts were stained with Acetic Carmine, dehydrated in an ethanol series and cleared in clove oil for subsequent mounting in Canada balsam. The specimens were deposited in the Invertebrate Collection of the National Museum of Natural History (USNM), Smithsonian Institution, Suitland, MD, including a neotype (USNM 1583310) and six paraneotypes (USNM 1583311-1583316). Taxonomic identification of *Pa. otobothrioides* was carried out with Dollfus (1966), and the classification and terminology of the larval stages follows Palm (2004) and Chervy (2002). The microtriches terminology follows Faliex et al. (2000) and Chervy (2009), with hamulate microtriches being oriented perpendicular to the surface of the tegument so that, unlike most other spinitriches, they appear to be laterally, rather than dorsoventrally, flattened. All morphological measurements are given in micrometers (μm) unless otherwise indicated.

### 2.2 Histological analyses

Live parasitized krill were randomly chosen on board from different sampling stations, fixed with Davidson's solution (Howard and Smith, 1983), placed in plastic cassettes and kept in dark during 48 h at room temperature. After this chemical treatment, all samples were preserved in 70% ethanol solution for histological analysis. In land-based laboratory, all specimens were dehydrated with successive series of ethanol concentrations (80, 90, and 96%) for 1 h at each concentration. Parasitized krill were embedded in Paraplast X-Tra at 54–56 °C fusion. Then plerocerci were cut into 4-mm longitudinal and transversal sections of whole specimens (Leica RM 2155 rotatory microtome) and stained for 6 min with Harris's hematoxylin and counterstained for 12 min with eosinphloxine (Sheenan and Hrapchak, 1973; Humason, 1979).

### 2.3 Scanning electron microscope observations

Surface ultrastructure of three entire and broken blastocysts with visible scolices, and dissected specimens with everted tentacles were examined under scanning electron microscope (SEM). Three specimens were dehydrated individually through alcohol dehydration series, dried with CO_2_ in a Polaron E3000 critical-point dryer. Each metacestode specimen was manipulated with watchmaker's tweezers under a stereoscope and mounted with a double-sided adhesive carbon tape onto SEM stubs. Then the stubs were coated with gold-palladium using a sputter coater (Polaron E5100) in an argon atmosphere and examined under a Hitachi S–3000N Scanning Electron Microscope at 20 Kv.

### 2.4 Genetic analysis

Total genomic DNA was extracted from ethanol-preserved parasites (blastocyst ~460 μm in diameter). Parasites were individually placed into 0.6 mL Eppendorf tubes and squashed by pressing it against the tube wall with a fine pipette containing 45–60 μL of 5% Chelex® 100. Then, proteinase K (15 mg/mL) was added followed by incubation for 45 min at 60 °C, boiled in a water bath for 8 min and centrifuged at 10 000 rpm for 5 min at 25 °C. Supernatant was used as template in subsequent PCR reactions.

The PCR amplification (T100™ Thermal Cycler BIO-RAD) of ssrDNA fragments (1989 bp) was carried out for 8 *Pa.* *otobothrioides* specimens. PCR was carried out using two universal primers, 1F 5′–AACCTGGTTGATCCTGCCAG–3′ and 1528R 5′–TGATCCTTCTGCAGGTTCACCTAC–3′ (Medlin et al., 1988). Additionally we used primers WormA 5′–GCGAATGGCTCATTAAATCAG–3'; WormB 5′–TTGTTACGACTTTTACTTCC–3′ and internal primers 1270F 5′–ACTTAAAGGAATTGACGG–3′ and 1270R 5′–CCG TCAATTCCTTTAAGT–3′ for full double-stranded coverage (Littlewood and Olson, 2001).

Cycling protocol for approximately 1989 bp partial ssrDNA with primers 1F and 1528R were as follows: denaturation for 5 min at 95 °C, followed by 35 cycles of 1 min at 95 °C, 1 min at 55 °C, 2 min at 72 °C; and 10 min extension at 72 °C. Cycling protocol for primers WormA and WormB, and 1270F and 1270R were as follows: denaturation for 2 min at 94 °C, followed by 40 cycles of 30 s at 94 °C, 30 s at 54 °C, 2 min at 72 °C, and 10 min extension at 72 °C.

The PCR mixtures comprised 0.5 μl of dNTPs (10 mM), 2.5 μl of buffer (15 mM MgCl_2_), 0.75 μl MgCl_2_ (50 mM), 2.5 μl of DMSO 5%, 2 μl of each primer (10 pmol/mL^−1^), and 1U of Taq polymerase (invitrogen 5U/μl). Final genomic DNA concentration varied from 150 to 300 ng/μl. Sterile water was added bringing the mixture to the final volume (25 μl).

Genomic DNA and PCR products were quantified with a Nanodrop Spectrophotometer (ND2000 Thermo scientific, BIO-RAD). 3 μl of the PCR products were subjected to electrophoresis in 1 × TBE buffer for 45 min at 80 V, loaded on 1% agarose gel stained with ethidium bromide to check DNA quality. The gels were photographed under a Chemi XRS Gel Documentation System (Multi-Imager, BIO-RAD).

Amplified PCR products were purified using the ExoSap purification kit (ExoSap-it, GE Healthcare, Piscataway, NJ, USA). Sequencing reactions were performed in a MJ Research PTC-225 Peltier Thermal Cycler using an ABI PRISM® BigDyeTM Terminator Cycle Sequencing Kits with AmpliTaq® DNA polymerase (FS enzyme) (Applied Biosystems). Single-pass sequencing was performed on each template using the two PCR primers and internal primers from both strands (Medlin et al., 1988; Littlewood and Olson, 2001; Littlewood et al., 2008). The fluorescent-labeled fragments were purified from the unincorporated terminators with BigDye® XTerminator™ purification protocol. The samples were resuspended in distilled water and subjected to electrophoresis in an ABI 3730xl sequencer (Applied Biosystems).

Editing, alignment and assembling of contiguous sequences were done with Bioedit version 7.2.5 (Hall, 1999). Sequence identity was checked using the Basic Local Alignment Search Tool (BLAST) (www.ncbi.nih.gov/BLAST/). Three assembled sequences were deposited at GenBank (http://www.ncbi.nlm.nih.gov/genbank/; under accession numbers: MH487651 – MH487653).

Phylogenetic analysis included sequences retrieved from GenBank including representatives of major families of all currently known trypanorhynch superfamilies. The accession number DQ642938 retrieved from GenBank was used for *Parachristianella indonesiensis* Palm, 2004 and not for *Shirleyrhynchus aetobatidis* (Shipley and Hornell, 1906) Beveridge and Campbell, 1998 (see Schaeffner, 2016; Haseli et al., 2017). Likewise, according with Schaeffner and Beveridge (2012) *Oncomegas australiensis* Toth, Campbell and Schmidt, 1992 (accsession DQ642957) and *Oncomegoides celatus* Beveridge and Campbell, 2005 (accsession DQ642934) were considered in the phylogenetic analysis for *Hispidorhynchus australiensis* (Toth, Campbell and Schmidt, 1992) Schaeffner and Beveridge, 2012 and *Oncomegas celatus* (Beveridge and Campbell, 2005), respectively. Phylogenetic analysis was performed using the Maximum Likelihood (ML) (Nei and Kumar, 2000) method based on the General Time Reversible model with gamma distributed with invariant sites (GTR + G + I). The model of evolution for phylogenetic analysis among Trypanorhyncha and genetic distance among Tentacularioidea were selected using JModeltest according to the Akaike Information Criterion (AIC). Phylogenetic relationships were evaluated using nonparametric bootstrap analysis (bootstrap values ≥ 70 were considered well supported), the tree with the highest log likelihood (−8800.1935) is shown (Felsenstein, 1985). Initial tree(s) for the heuristic search were obtained by applying the Neighbor-Joining method to a matrix of pairwise distances estimated using the Maximum Composite Likelihood (MCL) approach. A discrete Gamma distribution was used to model evolutionary rate differences among sites (5 categories (+G, parameter = 0.1602)). Diphyllideans *Ditrachybothridium macrocephalum* Rees, 1959, *Echinobothrium chisholmae* Jones and Beveridge, 2001, *Echinobothrium harfordi* McVicar, 1976 and *Echinobothrium* (=*Macrobothridium*) sp. were considered as outgroups to root the MCL tree. The analysis involved 61 nucleotide sequences. All positions containing gaps and missing data were eliminated. There was a total of 1748 positions in the final dataset. The phylogenetic tree was reconstructed using MEGA version 6.0 (Kumar et al., 2001).

## 3 Results

### 3.1 Infection pattern

Euphausiids community in the Gulf of California included 11 krill species from five distinct genera: *Euphausia diomedeae* Ortmann, 1884*, E. distinguenda* Hansen, 1911, *E. eximia* Hansen, 1911, *E. lamelligera* Hansen, 1911, *E. tenera* Hansen, 1905, *Nematoscelis difficilis* Hansen, 1911, *N. gracilis* Hansen, 1910, *Nematobrachion flexipes* (Ortmann, 1893), *Stylocheiron affine* Hansen, 1910, *S. carinatum* G. O. Sars, 1883 and *N. simplex*. *Paranybelinia otobothrioides* metacestodes infected only *N. simplex* with a prevalence ranging between 0.1 and 14.3% per oceanographic station. The highest infection levels were observed near the coast and mostly in the northern region of the Gulf of California. The intensity of infection was typically one cestode per krill, although co-infection with at least two other trypanorhynch species was also observed.

### 3.2 Morphology

**Fig. 1:**
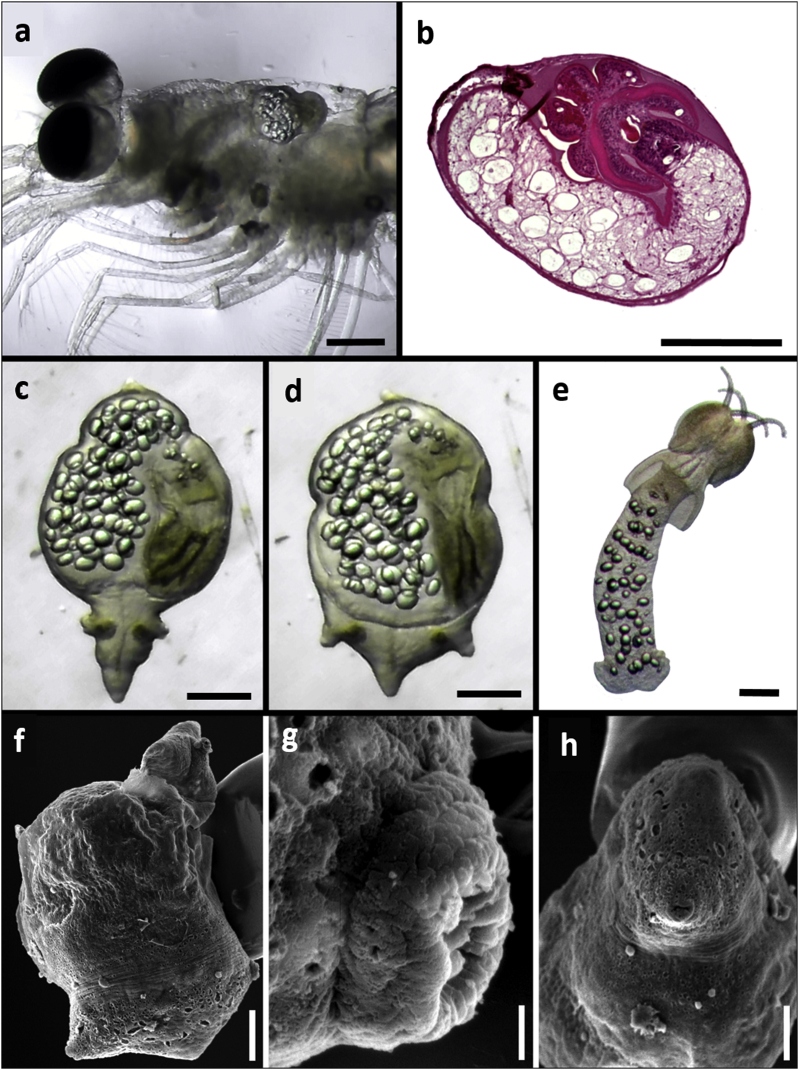
*Paranybelinia otobothrioides* parasitizing the subtropical krill *Nyctiphanes simplex* collected in the Gulf of California, Mexico. (A) Alive *Pa. otobothrioides* observed in the hemocoel of the host. (B) Histological section showing longitudinal view of *Pa. otobothrioides* inside the blastocyst. (C,D) Blastocyst containing the plerocercus of the same specimen, note the contraction (C) and expanding (D) movement of the blastocyst. (E) *Pa. otobothrioides* scolex dissected from the blastocyst. (F) Scanning electron microscope external view of the blastocyst, showing single distinct projectable centrally oriented porous-like terminal end (anterior), and 2 lateral and 1 central cone terminal projection (posterior end). (G,H) Detail of funnel openings at the anterior and posterior end. *Scale bars*: (A–F) 100 μm, (G) 5 μm, (H) 30 μm.

**Fig. 2:**
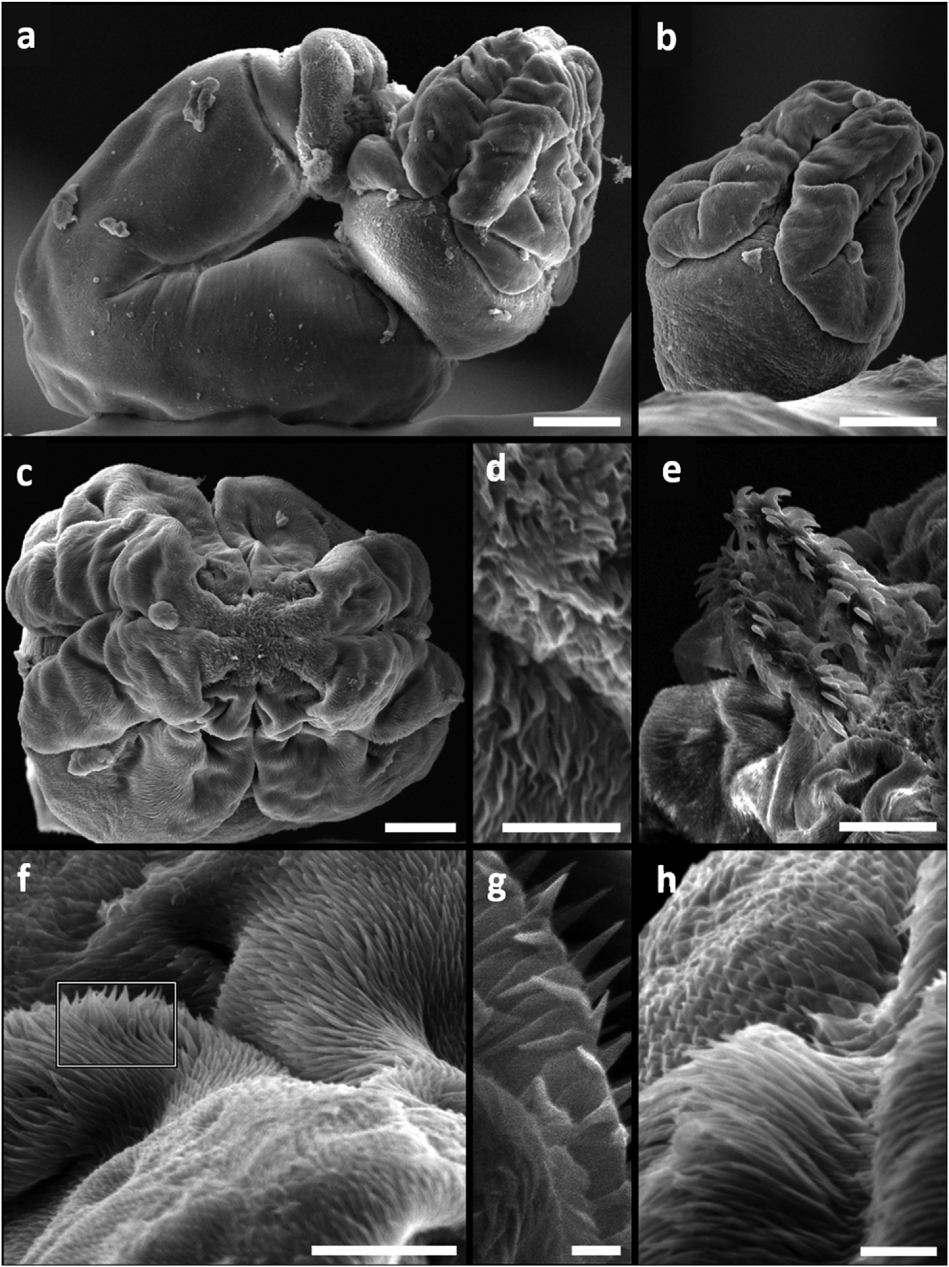
Surface ultrastructure of the scolex and tentacular armature of *Pa. otobothrioides*. (A) Dorso-ventral view of the scolex dissected from the blastocyst showing the distal bothrial surface without complete median separation. (B) Pedunculus scolecis showing 2 bothria with free lateral and posterior margins. (C) Apical view of the scolex bothrial surfaces. (D) Capilliform filitriches. (E) External tentacle surface, metabasal armature with solid uncinate hooks. (F) Distal bothrial surface showing (G) hamulate spinitriches and (H) lineate spinitriches. *Scale bars*: (A,C) 100 μm; (D–H) 50 μm.

The scolices of *Pa. otobothrioides* with surrounding blastocysts (n = 47) occurred in two microhabitats, mostly embedded in the hepatopancreas and rarely in the hemocoel of the host (Fig. 1A). Plerocerci infecting *N. simplex* were never observed encapsulated or freed from the blastocyst (Fig. 1A and B). The blastocyst appeared simple, consisting of a thin single-wall (thickness = 3) surrounding the craspedote scolex of *Pa. otobothrioides* (Fig. 1, Fig. 2, Fig. 4, Table 1). The scolex moved very slowly through rotatory movements inside the blastocyst contrasting with the digestive gland of the infected hosts (Fig. 1A). The scolex of *Pa. otobothrioides* shows a distinctive velum, free lateral and posterior margins of the bothria and a long appendix, with numerous rounded lipid-like filled droplets of variable diameter (36 ± 11.6) (Fig. 1A–E, Table 1). The plerocercus (blastocyst containing the scolex) showed constant peristaltic movements that typically began from the anterior part at a single distinct projectable centrally oriented porous-like terminal end (Fig. 1C–D, F, H), allowing to detect the parasite inside the host's hepatopancreas. Widening into the main part of the blastocyst including the larval scolex, the posterior part has two lateral and one central cone-like terminal projection (Fig. 1C–D, F). These projections are terminated with a protrusible porous-like structure (Fig. 1C–D, G).

**Fig. 3:**
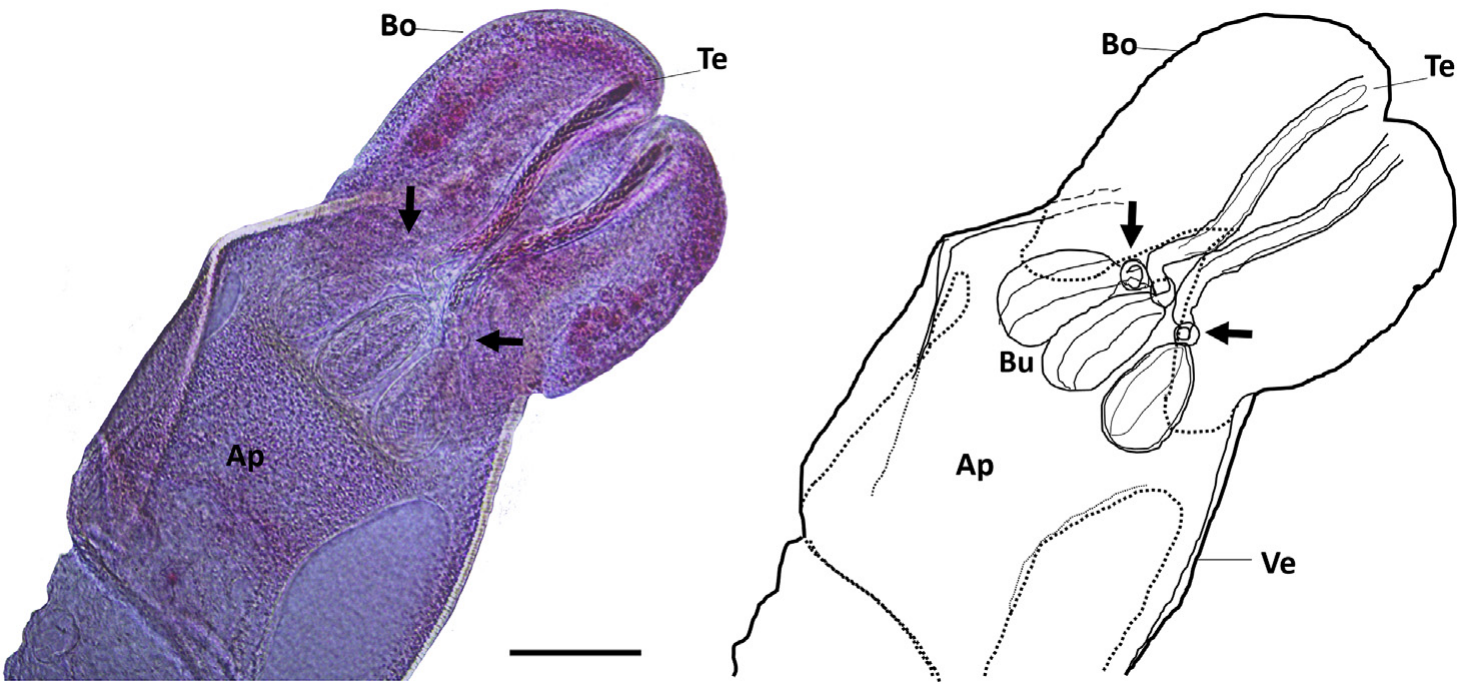
*Paranybelinia otobothrioides* anatomy showing details of the bothria (Bo), tentacles (Te), appendix (Ap), bulbs (Bu), and velum (Ve). The arrows show the muscular rings surrounding the tentacle sheaths. Scale = 60 μm.

**Table 1:** Measurements (μm) of *Paranybelinia otobothrioides* pleocercus parasitizing the subtropical. krill *Nyctiphanes simplex*. SD = Standard deviation.

Structure	Morphological characters	Mean	Range	SD	n
Blastocyst	Length	466.8	303.5–686	129	25
** **	Width	300.5	203.4–470.4	89.2	27
** **	Wall thickness	3	1.4–5.6	1	32
** **	Lateral opening diameter	10.5	9.5–12.5	1.4	4
** **	Posterior openning diameter	4.4	4.4		1
Scolex	Length	689.8	443.6–940.4	146.7	22
	Width	205.4	205.4		1
	Pars bothridialis	143.4	111.6–192	21	25
	Bothria length	131.6	106.5–166.3	16.2	30
	Bothrial width at ant. margin	56.2	45.9–72	7.8	18
	Bothrial width at base	158.5	111.7–206.6	25.4	25
	Pars vaginalis	108.7	65–127.2	17.7	17
	Pars bulbosa	66.2	42.6–89.5	14	23
	Bulb length	60.6	42.3–79.2	12.5	20
	Bulb width at base	34.2	21.7–44.5	7.9	17
	Bulb width at anterior margin	16.6	10.2–23.1	3.7	11
	Pars post bulbosa	78.0	37.4–105.6	14.8	16
	Pedunculus scolecis	229	127.8–297.6	43.2	29
	Velum length	84.4	43.2–122.4	16.7	31
	Velum width	169.2	96.5–225.4	32.7	27
	Velum width at base	113.3	64–166.6	28.7	16
	Velum maximum thickness	20.8	15.6–31.2	6.4	5
	Appendix length	542.9	310.8–793.8	132	12
	Appendix anterior width	122.5	89.1–145	20	7
	Appendix posterior width	168.4	97.7–274	66.3	12
	Diameter of granules	36.2	15–55	11.6	26
	Bothrial groove diameter	4.5	3.2–7.2	1.9	4
Tentacles	Tentacle length	96.7	49.3–152.5	37.4	13
	Tentacle width at distal end	9.7	6.3–15.5	3.5	8
	Tentacle width at base	10.4	8.7–11.7	1.2	4
	Muscular rings surrounding the tentacle sheaths	Yes			
Hooks	Hook length	7.5	4.5–9.5	1.1	16
	Hook base length	4.6	2.6–8.6	1.4	21
	Hook height base-tip	4.9	2.83–7.4	1.1	19

Scanning electron microscopy (SEM) observations of the blastocyst-dissected plerocercus of *Pa. otobothrioides* showed details of the scolex, tentacular armature, and surface ultrastructure (Fig. 2A–C). Measurements of the blastocyst and the small-sized craspedote scolex are given in Table 1. Two large bothria cover over half of the scolex length and extend into the pars bulbosa scolecis (Fig. 2A–C). In lateral view, the scolex shows a single large bothrium with free lateral and posterior margins (Fig. 2A). At the centre of the distal bothrial surface the bothrium forms two lateral bulges, looking like a distinct separation of the bothrium into two bothria. However, the middle and anterior part covered with the typical hamulate spinitriches demonstrates that the separation of the two bothria into four is not complete (Fig. 2A, F). The lateral and frontal view of the bothria showed distinct separation of the two bothria and illustrate the dorsal/ventral surfaces (Fig. 2B and C). The tentacular armature is homeoacanthous homeomorphous with uncinated solid hooks (Fig. 2E, Table 1) and lacks a characteristic basal armature. Tentacle sheaths are straight. Muscular rings around the basal part of tentacle sheaths are present and the retractor muscles originate at the basal part of bulbs (Fig. 3A and B).

### 3.3 Surface ultrastructure

**Fig. 4:**
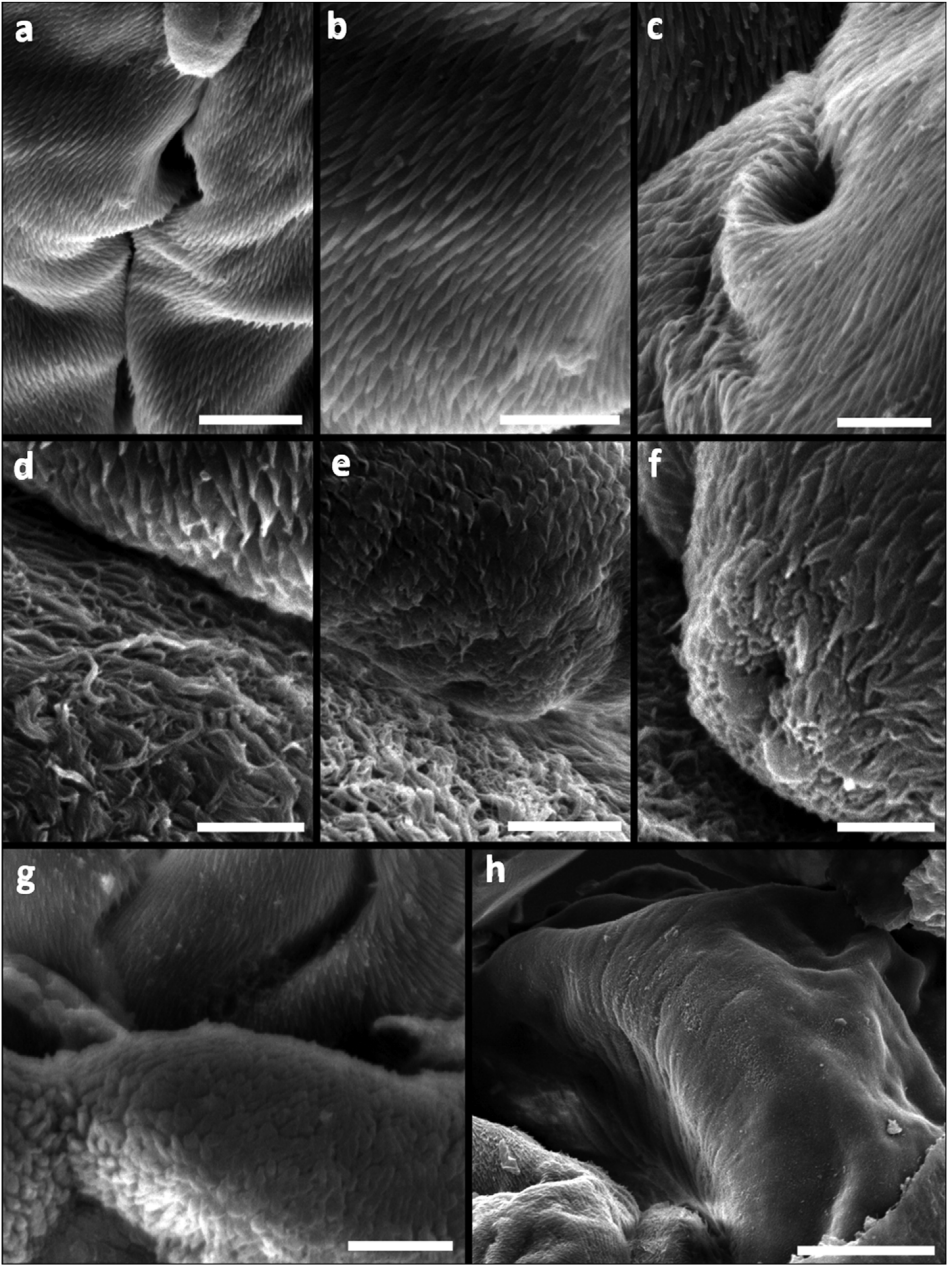
Surface ultrastructure of the scolex of *Paranybelinia otobothrioides*. (A) Anterolateral bothrial margins showing the distribution pattern of hamulate spinitriches with extended bases. (B,C) Hamulate spinitriches along the bothrial margins and on the distal bothrial surface. (D) Lineate spinitriches on the posterior margins of the bothria. (E) Tegumental groove at the posterior part of the bothria surrounded by lineate spinitriches and capilliform filitriches on the pars postbulbosa (also on pars vaginalis and bulbosa). (F) Tegumental groove at the posterior part of the bothria showing similar spinitriches (lineate or hamulate) to the surrounding bothrial surface. (G) Posterior end of appendix. (H) Appendix covered with papilliform filitriches. *Scale bars*: A, C, 100 μm; D-H, 50 μm.

The scolex revealed four subtypes of microtriches: capilliform and papilliform filitriches, and hamulate and lineate spinitriches. Capilliform filitriches (length range = 1.1–2.8, mean width = 0.22) cover the apical scolex region (Fig. 2D). Hamulate spinitriches with extended bases (length range = 1.5–2.5, mean width = 0.7) cover the entire distal bothrial surface (Fig. 2F–H), including the lateral bothrial margins (Fig. 4A–C). These hamulate spinitriches appear lineate at the posterior margins of the bothria (Fig. 4D). The scolex peduncle is covered with capilliform filitriches (length range = 2–2.4, mean width = 0.2) (Fig. 4D, E, G), and papilliform filitriches adorn the entire appendix (Fig. 4H). Tegumental grooves (2 ± 0.5 diameter) (formerly called bothrial pits) are evident at the posterior bothrial margins, two on each bothrium (Fig. 4E and F). No distinct spinitriches were observed covering this scolex structure, and they show similar hamulate or lineate spinitriches compared with the surrounding bothrial surface.

### 3.4 Phylogenetic placement of Paranybelinia otobothrioides

The 1989 bp concatenated fragment of the small subunit rDNA (ssrDNA) of *Pa. otobothrioides* showed the nucleotide composition proportion of T = 25.4, C = 22, A = 23.9, G = 28.7, and G + C = 50.7%. The analyzed molecular data set consisted of 58 trypanorhynch sequences, including 11 species belonging to the Tentaculariidae and 2 species belonging to the Rhinoptericolidae, both families considered to be related with the Paranybeliniidae. The aligned 18S partition comprised 1913 positions of which 525 (26%) positions were variable and 400 (20%) were parsimony informative.

**Fig. 5:**
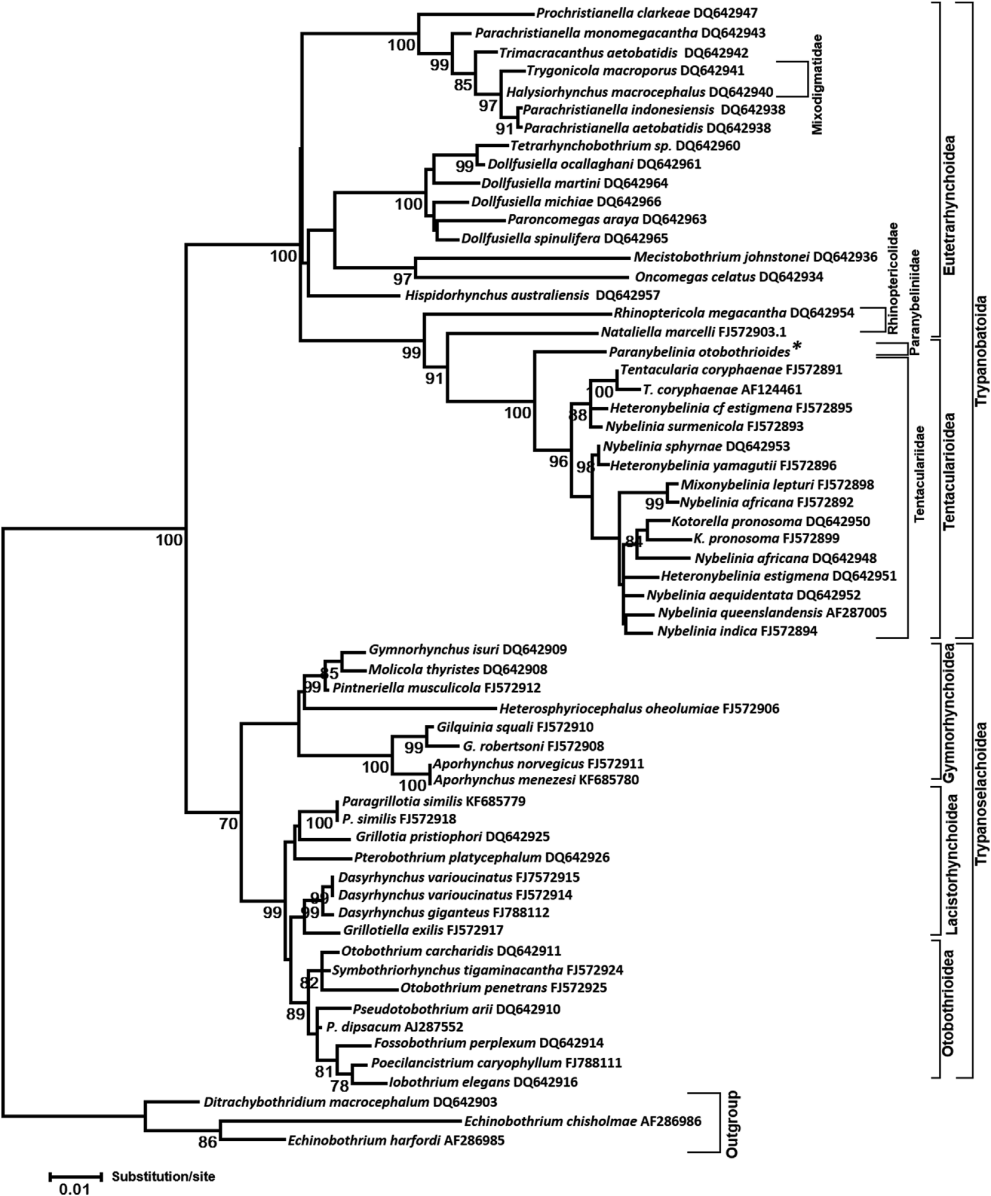
Maximum Likelihood tree under the General Time Reversible model of selected trypanorhynchs including *Paranybelinia otobothrioides*. Major families, superfamilies, and orders are indicated. Numbers on the branches show nodal support.

The Maximum likelihood analysis of the ssrDNA was largely congruent with the known topology of trypanorhynch cestodes (see Palm et al., 2009; Olson et al., 2010) with two major clades, the suborders Trypanoselachoida and Trypanobatoida (bootstrap values range 70–100). Inside the suborder Trypanobatoida, the genera *Prochristianella*, *Parachristianella*, *Trimacracanthus* (Eutetrathynchiidae) together with *Trygonicola* and *Halysiorhynchus* (Mixodigmatidae) formed a high supported clade (bootstrap 100%). This group placed sister to a weak supported clade (bootstrap <70%), consisting of *H. australiensis*, *Mecistobothrium johnstonei* (Southwell, 1929) Beveridge & Campbell, 1998 and *Oncomegas celatus* (Beveridge and Campbell, 2005) Schaeffner and Beveridge, 2012 [ = *Oncomegoides celatus*]) and the well supported clade formed by the *Tetrarhynchobothrium* Diesing, 1854, *Dollfusiella* Campbell and Beveridge, 1994, and *Paroncomegas* Campbell, Marques and Ivanov, 1999 (bootstrap 100%) (Fig. 5).

The superfamily Tentacularioidea was represented in a distinct major clade (bootstrap 100%) (Fig. 5). The phylogenetic analysis placed *Pa. otobothrioides* in a clade with high nodal support (bootstrap 100%) as sister taxon to the tentaculariids. The species *Rhinoptericola megacantha* Carvajal and Campbell, 1975 and *Nataliella marcelli* Palm, 2010 (Rhinoptericolidae), despite considered as members of the Eutetrarhynchoidea, were placed as sister taxon to the superfamily Tentacularioidea (i.e., Tentaculariidae and Paranybeliniidae) with strong nodal support (bootstrap > 90).

Estimates of evolutionary divergence based on Kimura-2-parameters corrected distances (K2P) and the number of base differences per site between sequences were calculated between the Tentacularioidea (Table 2), including the Tentaculariidae and the Paranybeliniidae. *Paranybelinia otobothrioides* showed no intraspecific genetic variation. Estimates of K2P between *Pa. otobothrioides* and the 11 tentaculariid species ranged from 0.027 to 0.039 (average = 0.033) and the number of base differences per sequence (nd) ranged from 44 to 62. Inside the Tentaculariidae, the genetic distance resulted in a considerably lower K2P range from 0.003 to 0.03 (average = 0.018) with the nd ranging from 5 to 49. Of the analyzed Tentaculariidae, *Tentacularia coryphaenae* Bosc, 1802 was the closest species to *Pa. otobothrioides* (K2P = 0.027, nd = 44), whilst *Nybelinia africana* Dollfus, 1960 showed to be the most divergent sequence (K2P = 0.039, nd = 62) (Table 2).

**Table 2:** Kimura 2-parameters (K2P) genetic distances and nucleotide differences among Tentacularioidea: *Paranybellinia otobothrioides* (Paranybeliniidae) respect to species of the family Tentaculariidae based on ssrDNA.

	Species	1	2	3	4	5	6	7	8	9	10	11	12	13	14	15	16
1	*Paranybelinia otobothrioides*		56	49	45	60	61	56	52	59	62	53	45	57	45	53	44
2	*Heteronybelinia estigmena*	0.035		37	28	23	31	21	16	20	32	16	27	20	37	45	36
3	*Heteronybelinia cf estigmena*	0.031	0.023		17	38	41	39	30	42	34	30	16	34	8	23	14
4	*Heteronybelinia yamagutii*	0.028	0.017	0.010		31	37	25	21	30	32	24	5	27	19	28	19
5	*Kotorella pronosoma*	0.038	0.014	0.023	0.019		25	30	20	29	30	19	32	25	37	47	38
6	*Kotorella pronosoma*	0.038	0.019	0.025	0.023	0.015		37	28	37	35	29	35	35	40	47	38
7	*Mixonybelinia lepturi*	0.035	0.013	0.024	0.015	0.018	0.023		22	5	39	25	27	29	41	46	37
8	*Nybelinia aequidentata*	0.033	0.010	0.018	0.013	0.012	0.017	0.013		25	27	13	22	17	30	37	28
9	*Nybelinia africana*	0.037	0.012	0.026	0.018	0.018	0.023	0.003	0.015		40	26	30	30	42	49	40
10	*Nybelinia africana*	0.039	0.020	0.021	0.020	0.018	0.022	0.024	0.017	0.025		27	33	34	31	47	38
11	*Nybelinia indica*	0.033	0.010	0.018	0.015	0.012	0.018	0.015	0.008	0.016	0.017		23	14	31	40	31
12	*Nybelinia sphyrnae*	0.028	0.016	0.010	0.003	0.020	0.021	0.016	0.013	0.018	0.020	0.014		26	18	31	22
13	*Nybelinia queenslandensis*	0.036	0.012	0.021	0.017	0.015	0.022	0.018	0.010	0.018	0.021	0.008	0.016		34	44	35
14	*Nybelinia surmenicola*	0.028	0.023	0.005	0.012	0.023	0.025	0.025	0.018	0.026	0.019	0.019	0.011	0.021		20	11
15	*Tentacularia coryphaenae*	0.033	0.028	0.014	0.017	0.029	0.029	0.029	0.023	0.030	0.029	0.025	0.019	0.027	0.012		9
16	*Tentacularia coryphaenae*	0.027	0.022	0.008	0.012	0.023	0.023	0.023	0.017	0.025	0.023	0.019	0.013	0.022	0.007	0.005	

## 4 Discussion

The present study is the first detailed description of both anatomy and external morphology of the *Pa. otobothrioides* blastocyst and plerocercus and the first larval trypanorhynch from krill that it is placed in a phylogenetic context. We show that *Pa. otobothrioides* has a broad geographical range of distribution inhabiting tropical waters in the East coast of the Atlantic (Cape Verde Islands, West coast of Africa) (Dollfus, 1966) and subtropical waters in the East Pacific Ocean (Gulf of California) (the present study).

### 4.1 Host range

The most recent review on parasites infecting euphausiids (krill) listed five orders and six families of cestodes (Gómez-Gutiérrez et al., 2017). However, larval trypanorhynch cestodes infecting krill are difficult to identify to species level mainly because the scolex is enclosed in a thin membrane or a simple protective blastocyst and also by the lack of characters typically observed only in the adult stage (i.e., strobila characters). Thus, their identification to species level is difficult to achieve by using morphological features alone. Trypanorhynch cestodes were reported infecting the krill *N. simplex* (Gómez-Gutiérrez et al., 2010) and *Euphausia americana* Hansen, 1911 (González-Solís et al., 2013) from Mexican waters. These two reports highlighted new host and geographical records of infection, but global cestode species richness (component community) that parasitize krill as intermediate hosts remain underestimated. To date, from the helminths infecting euphausiids, *Nybelinia surmenicola* Okada in Dollfus (1929) and *Ps. odontacantha* are the only trypanorhynchiid cestodes identified so far to species level. *Nybelinia surmenicola* shows low host specificity being so far recorded parasitizing the euphausiids *Thysanoessa longipes* Brandt, 1851, *T. inermis* (Kumar et al., 2001), *T. raschi* (M. Sars, 1864), *Euphausia pacifica* Hansen, 1911 and *Nematoscelis* sp., while *Ps. odontacantha* has been recovered only from *E. recurva* (Shimazu, 1982, 2006).

Although we observed morphometric variability in the scolex of *Pa. otobothrioides*, overall measurements were consistent with the morphology of the specimens previously reported in Dollfus (1966). Similarly, morphometric variation observed in the blastocyst total length and total body size of *Pa. otobothrioides* suggests that ontogenetic changes occur inside the host. Additionally, high prevalence of *Pa. otobothrioides* infecting *N. simplex* and its absence in other krill species from the same sampling sites supports that this species functions as a required second intermediate host in the life cycle of this trypanorynch cestode in the Gulf of California and suggests a zooplankton feeding elasmobranch as final host.

### 4.2 The blastocyst of Pa. otobothrioides and its systematic importance

According to Dollfus (1966) *Pa. otobothrioides* and *Ps. odontacantha* showed distinctive morphological characters, i.e., homeoacanth tentacular armature, two bothria with the so called “bothrial pits” and the absence of blastocysts that led to the erection of an own family Paranybeliniidae to include these monotypic species. Shimazu (1982) recovered *Ps. odontacantha* infecting *E. recurva* from the East China Sea. This author highlighted that the metacestodes infecting krill lacked a blastocyst supporting findings provided in Dollfus (1966). Nevertheless, Shimazu (2006) pointed out that metacestodes of *Ps. odontacantha* (published in Shimazu, 1982) could have occurred in a blastocyst, though no evidence was provided. More recently, Palm (2008) described the surface ultrastructure of the scolex of *Ps. odontacantha* based on the type material deposited in the collection of the Museum National dʹHistorie Naturelle, Paris, France. However, since these trypanorhynch species had not been recovered after Dollfus (1966) and Shimazu (1982), morphological re-examination of *Pa. otobothrioides* and the formation of a blastocyst in both species remained to be confirmed.

In the present study, we show that the freshly collected specimens of *Pa. otobothrioides* occur enclosed in a blastocyst that could only be opened by dissection with acupuncture needles. The single layer tissue blastocyst containing the scolex was found inside the hepetopancreas of the host. This finding demonstrates that *Pa. otobothrioides* and most likely *Ps. odontacantha* (Shimazu, 1999, 2006) develop within a simple blastocyst when infecting their intermediate hosts, the euphausiids *N. simplex* parasitizing *Pa. otobothrioides* and *E. recurva* parasitizing *Ps. odontacantha*. Four pori in *Pa. otobothrioides*’ blastocyst more likely allow direct interaction between the coelomic fluid of the host and the inner part of the blastocyst surrounding the scolex, suggesting a possible feeding and/or excretory function. After opening the blastocyst, the scolex moved freely and resembled the typical plerocercoid stages known from the related tentaculariids. It is interesting to note that *Tentacularia coryphaenae*, one of the most closely related tentaculariid to *Pa. otobothrioides* in the present study, was earlier reported in a skinny “translucent host capsule” shown in Fig. 1 in Palm et al. (2007), and also Shimazu (1999) documented that the plerocercoids of *N. surmenicola* occur in a translucent simple blastocyst inside the temperate euphausiid *E. pacifica*.

The lack of a blastocyst was considered a plesiomorphic character (Hoberg et al., 1997; Beveridge et al., 1999). According to Palm (1997), its presence is a convergent development within the superfamilies of trypanorhynchs (absent in the Tentacularioidea and Gymnorhynchoidea, and present in the Otobothrioidea, Lacistorhynchoidea, and Eutetrarhynchoidea). Therefore, the lack or presence of a blastocyst was used to distinguish the trypanorhynch taxa at the family level only. However, when the species description was based only on adult specimens, the incipient knowledge of their early larval development infecting intermediate/paratenic hosts lead to tentatively assignment of some genera in superfamilies and families despite not knowing if their larvae have a blastocyst. In this context, the systematic position of *Pa. otobothrioides* has been unstable and widely debated allocating it within the superfamilies Tentacularioidea and the Otobothrioidea. Schmidt (1986), Campbell and Beveridge (1994) and Beveridge et al. (1999) based on a plerocercoid lacking a blastocyst and the presence of a homeoacanthous armature, affiliated (with cladistics) the genera *Paranybelinia* and *Pseudonybelinia* close to *Kotorella* and *Nybelinia* belonging to the family Tentaculariidae (Tentacularioidea). Although Shimazu (1999) stated that the tentaculariid *N. surmenicola* is enclosed in a simple blastocyst or bladder and infects the digestive gland of the krill *E. pacifica*, it was supposed that the superfamily Tentacularioidea lacks a blastocyst during their ontogeny (Dollfus, 1929; Palm and Walter, 2000; Palm, 2008). Herewith, a blastocyst has been observed in members of all five superfamilies Otobothrioidea, Lacistorhynchoidea, Gymnorhynchoidea, Eutetrarhynchoidea, and Tentacularioidea (Shimazu, 1999, 2006, present study).

Additionally, strong hyaline cysts surrounding plerocerci, the latter also complex multilayered or even merocercoids, have been recorded in lacistorhynchoid, otobothriod (e.g. Palm and Overstreet, 2000) and gymnorhynchoid trypanorhynchs (Palm, 2004). Mattis (1986) demonstrated that *Prochristianella hispida* (Linton, 1890) (Eutetrarhynchoidea) develops a different type of blastocyst in respect to *Poecilancistrum caryophyllum* (Diesing, 1850) (Otobothrioidea). The morphological features observed in the merely simple single-layered blastocyst including the plerocercoid scolex of *Pa. otobthrioides* are so far unique and can be easily distinguished from any other trypanorhynch. This suggests that in the classification of the trypanorhynchs, the blastocyst morphology and its development instead of the solely presence/absence of the blastocysts will add more taxonomic resolution at the family level. However, further morphological characterizations of blastocysts within families and genera are needed in order to evaluate the level of taxonomic precision and autapomorphies.

### 4.3 Surface ultrastructure of the scolex

The so called bothrial pits is a remarkable characteristic attributed to *Ps. odontacantha* and *Pa. otobothrioides* (Dollfus, 1966), both Paranybeliniidae. Based on the presence of these structures, both species were related close to the superfamily Otobothrioidea (Palm, 1995, 1997, 2004). However, Jones (2000), Palm et al. (1998, 2000) and Palm and Overstreet (2000) found that the bothrial pits of the Pseudotobothriidae and Otobothriidae have microtriches distinctly different to those of the surrounding bothrial surfaces. On the contrary, Palm (2008) could distinguish that the pit-like structures present at the proximal end of the bothria of *Ps. odontacantha* have microtriches that cannot be distinguished from those covering the remainder of the bothrial surface. Palm (2008) formally termed these structures as tegumental grooves, and the presence of hamulate spinitriches differentiate them from the bothrial pits of the superfamily Otobothrioidea. The specimens of *Pa. otobothrioides* of the present study showed tegumental grooves with hamulate spinitriches alike those covering the bothrial surface. This finding is congruent with the presence of tegumental grooves found in *Ps. odontacantha*, showing that these structures are a distinctive taxonomic character shared in the family Paranybeliniidae and suggesting its autapomorphy.

Consistent general microthrix patterns on the bothrial surfaces and the scolex peduncle have shown to be useful characters differentiating between trypanorhynch taxa ranging from species to family levels (Palm, 2008). For instance, cestodes of the family Tentaculariidae typically show filitriches on the scolex peduncle, and a combination of hamulate and lineate spinitriches with characteristic internal ultrastructure along the bothrial borders (Chervy, 2009). Additionally, hamulate combined with lineate spinitriches have been recorded only within the genera *Nybelinia*, *Heteronybelinia* and *Kotorella* (Palm, 1995; Jones and Beveridge, 1998; Palm and Overstreet, 2000). Likewise, tentaculariids lack capilliform filitriches on the posterior part of the appendix (Palm, 2000, 2008). In contrast, cestodes of the family Otobothriidae can be distinguished by possessing pectinate spinitriches on the bothrial surface. The distal bothrial surface and its borders have microtriches with digitiform projections varying from bidentate, hexadentate to palmate, whereas the entire scolex can be covered with spinitriches (Palm et al., 2000; Palm, 2008). The external surface ultrastructure of *Ps. odontacantha* showed the characteristic hamulate and lineate spinitriches combined with capilliform and papilliform filitriches from the tentaculariids (Palm, 2008). We herewith demonstrate that the surface ultrastructure of *Pa. otobothrioides* exhibits the same microtriches types and distribution pattern than its relative *Ps. odontacantha* together with similar scolex features. Excepting the occurrence of the hamulate spinitriches covering the entire bothrial surface and the absence of filitrich (capilliform) microtriches on the proximal part of the appendix, the microthrix pattern observed in *Pa. otobothrioides* and *Ps. odontacantha* is most consistent with the surface ultrastructure exhibited by the family Tentaculariidae and not the Otobothriidae. However, *Pa. otobothrioides* and *Ps. odontacantha* possess two dorsal-ventral bothria partially divided and not completely separated from each other easily distinguished from the tentaculariids that either possess 4 elongate or triangular clearly separate bothria.

### 4.4 Phylogenetic placement of Paranybelinia otobothrioides

The ssrDNA analysis of the trypanorhynch relationships including the new sequence of *Pa. otobothrioides* was consistent with results previously reported in studies based on concatenated ribosomal markers (e.g. Palm et al., 2009). The superfamily Eutetrarhynchoidea appeared paraphyletic. It comprises four clades, remaining the family relationships within the superfamily unresolved. The *Prochristianella*/*Parachristianella/Trimacracanthus/*clade together with the nested Mixodigmatidae (*Trygonicola*/*Halysiorhynchus*) combines genera with 2 and 4 bothria, the former with a heteroacanthous typical and the Mixodigmatidae with a poeciloacanthous armature. The species *Hispidorhynchus australiensis* represents a second clade with 2 bothria and a heteroacanthous homeocanthous armature sister to the *Tetrarhynchobothrium/Dollfusiella*/*Paroncomegas* clade and the phylogenetically derived clade *Mecistobothrium/Oncomegas* that unite heteroacanthous and homeocanthous species with 2 bothria.

A further trypanorhynch clade included species with 4 bothria before placement of *Pa. otobothrioides* in the present study (Palm et al., 2009). That phylogenetic hypothesis placed the heteroacanthous *Rhinoptericola megacantha* with 4 bothria and prebulbar organs (Rhinoptericolidae) together with *Nataliella marcelli* Palm, 2010 (4 bothria, homeoacanthous, prebulbar organs, also Rhinoptericolidae in Palm, 2010) in a clade with the mainly homeoacanthous tentaculariids, intermediate between a subgroup of the paraphyletic ‘eutetrarhynchoids’ and the tentacularioids (Palm et al., 2009). *Paranybellinia otobothrioides* positioned inside the clade with the Rhinoptericolidae (Eutetrarhynchoidea) and the Tentaculariidae (Tentacularioidea). However, with its characteristic scolex morphology, surface ultrastructure and the muscular rings around the tentacle sheaths instead of prebulbar organs, *Pa. otobothrioides* is clearly affiliated with the Tentacularioidea and not the Eutetrarhynchoidea.

Having 2 bothria instead of 4, *Pa. otobothrioides* forms a distinct clade sister to the species representing the family Tentaculariidae. This result concurs with the hypothesis originally proposed by cladistic analyses which demonstrated a close relationship of the family Paranybeliniidae with the Tentaculariidae (Campbell and Beveridge, 1994; Beveridge et al., 1999); and with the transfer of the Paranybeliniidae into the Tentacularioidea based on scolex morphology and surface ultrastructure as suggested by Palm (2008). The sister-group relationship between *Pa. otobothrioides* and the Tentaculariidae also strongly supports the monophyly of the superfamily Tentacularioidea as proposed by Palm et al. (2009).

Genetic distance has been used as an important indicator for species discrimination (e.g. Haseli et al., 2017), ranging between allopatric conspecifics in members of the Eutetrarhynchidae (0.22%–1.66%), Tentacularioidea (0.00%–0.11%), Gymnorhynchoidea (0.00%–0.08%) and Lacistorhynchoidea (0.00%–0.33%) (Palm et al., 2007, 2009). To date, there is no genetic distance threshold to distinguish family boundaries inside the Trypanorhyncha. However, the genetic distance values observed between *Pa. otobothrioides* and the Tentaculariidae (average K2P = 0.033 [3.3%]; Table 2) could be consistent with that observed among monophyletic superfamiles. The low degree of divergence of the ssrDNA analyzed fragment most likely reflects species that are delimited by more subtle morphological traits. Based on the average K2P observed between the Tentacularioidea, which is supported by several differences in morphological features of *Pa. otobothrioides* (e.g. 2 bothria [not entirely split into 4 bothria, see Fig. 2a–c, Fig. 3a,] with free lateral and posterior margins, 4 posterior tegumental grooves, and hamulate mictotriches on the entire distal bothrial surface) together with the characteristic blastocysts inside euphausiids as second intermediate hosts, we therefore maintain the Paranybeliniidae as an own family within the Tentacularioidea, necessitating emendation of the superfamily diagnosis in the most recent classification by Palm (2004).

## Conflicts of interest

The authors declare that there are no conflicts of interest.
